# An improved method for RNA isolation and cDNA library construction from immature seeds of *Jatropha curcas *L

**DOI:** 10.1186/1756-0500-3-126

**Published:** 2010-05-05

**Authors:** Jatinder Singh Sangha, Keyu Gu, Jatinder Kaur, Zhongchao Yin

**Affiliations:** 1 Temasek Life Sciences Laboratory, 1 Research Link, the National University of Singapore, Singapore 117604, Republic of Singapore; 2 Department of Environmental Sciences, Nova Scotia Agricultural College, Truro, B2N5E3, NS, Canada; 3 Department of Plant and Animal Sciences, Nova Scotia Agricultural College, Truro, B2N5E3, NS, Canada

## Abstract

**Background:**

RNA quality and quantity is sometimes unsuitable for cDNA library construction, from plant seeds rich in oil, polysaccharides and other secondary metabolites. Seeds of jatropha (*Jatropha curcas *L.) are rich in fatty acids/lipids, storage proteins, polysaccharides, and a number of other secondary metabolites that could either bind and/or co-precipitate with RNA, making it unsuitable for downstream applications. Existing RNA isolation methods and commercial kits often fail to deliver high-quality total RNA from immature jatropha seeds for poly(A)^+ ^RNA purification and cDNA synthesis.

**Findings:**

A protocol has been developed for isolating good quality total RNA from immature jatropha seeds, whereby a combination of the CTAB based RNA extraction method and a silica column of a commercial plant RNA extraction kit is used. The extraction time was reduced from two days to about 3 hours and the RNA was suitable for poly(A)^+ ^RNA purification, cDNA synthesis, cDNA library construction, RT-PCR, and Northern hybridization. Based on sequence information from selected clones and amplified PCR product, the cDNA library seems to be a good source of full-length jatropha genes. The method was equally effective for isolating RNA from mustard and rice seeds.

**Conclusions:**

This is a simple CTAB + silica column method to extract high quality RNA from oil rich immature jatropha seeds that is suitable for several downstream applications. This method takes less time for RNA extraction and is equally effective for other tissues where the quality and quantity of RNA is highly interfered by the presence of fatty acids, polysaccharides and polyphenols.

## Background

Efficient isolation of high quality and quantity of total RNA from plants is highly desirable for the construction of a good-quality cDNA library. Several commercial reagents and kits are available for isolating RNA from plants (e.g., Trizol, Gibco-BRL Life Technologies; RNeasy plant kit, QIAGEN), but they are not always effective for all plant tissues, particularly for developing seeds of oil rich plants. Seeds from oilseed plants contain high levels of extractable lipids, polysaccharides and phenols and many other secondary metabolites that could interfere with RNA extraction, by degrading or co-precipitating with the extracted RNA [1-3]. Thus most RNA isolation methods either result in very low yields of RNA or form complexes with these contaminants resulting in low quality poly(A)^+ ^RNA unsuitable for first strand cDNA synthesis and RT-PCR [4]. Such undesirable outcomes prompted researchers to develop new improved protocols for RNA extraction from several recalcitrant plant tissues [5-8].

Jatropha (*Jatropha curcas *L.) is a small tropical, woody plant belonging to the Euphorbiaceae family and is found in many tropical and subtropical countries. Even though the seeds of jatropha are highly toxic due to the protein 'curcin' and phorbol esters [9], almost all parts of this plant have been utilized, either in insecticides, green manure, soap making, medicine, just to name a few [10,11]. Jatropha seeds contain about 40% of oil enriched with both saturated [palmitic acid (16:0, 14.1%) and stearic acid (18:0, 6.7%)] and unsaturated [oleic acid (18:1Δ^9^, 47.0%) and linoleic acid (18:2Δ^9,12^, 31.6%)] fatty acids [12-14]. During maturity, large amounts of fatty acids/lipids, several toxic compounds and other secondary metabolites are found in jatropha seeds particularly in four to five-week-old seeds [15]. These compounds are known to interfere directly with nucleic acid extraction from different biological samples [5,8]. In order to isolate high-quality intact RNA from such tissues, removal of these contaminating substances is necessary to prevent them from binding to nucleic acids [3,4]. With increasing demand for biofuel production, oil rich crops like jatropha are being explored through biotechnology. Information on the genome of jatropha is emerging and the functions of individual genes are being determined [10,16]. Isolation of high-quality RNA from immature jatropha seeds will be useful to construct an efficient cDNA library for understanding the molecular basis in seed oil improvement.

Cetyltrimethylammonium bromide (CTAB) based method was developed for RNA extraction from tissues containing high levels of polysaccharides and phenols [17]. The protocol is good for several recalcitrant plant tissues, but not always effective for the others, and therefore modified and improved as per requirement [5,8,18]. RNA extraction from jatropha developing seeds is difficult due to its complex properties. The published information on jatropha RNA isolation methods is very limited [19]. We tried several methods without successfully extracting a good amount of intact RNA from the seeds. We modified the CTAB RNA extraction method and combined it with silica column of RNeasy^® ^Plant Mini Kit (Qiagen, Germany) to develop a simple, quick and efficient protocol for isolating RNA from immature jatropha seed. The RNA extracted with this method was good for several downstream applications such as cDNA library construction, RT-PCR, gene isolation and Northern blot analysis. This method was equally good for seeds of mustard (*Brassica spp.*) and starchy rice (*Oryza sativa*).

## Methods

### Plant tissue collection

Immature jatropha (*Jatropha curcas*) seeds, at 4-5 weeks after fertilization were selected for total RNA extraction. Rice (*Oryza sativa*) seeds 21 days after fertilization and mustard (*Brassica spp.*) seeds 20 days after fertilization were also used. Before grinding, the kernels of immature jatropha seeds were separated from the seed shell, whereas for rice seed husks were removed using sterilized scissors or forceps and stored at -80°C until use. Mustard seeds were removed from pods and used as such.

### Total RNA extraction method

About 0.5 g of each seed sample was ground in liquid nitrogen using oven baked RNase-free mortar and pestle and the seed powder was then transferred to a pre-chilled 50-mL polypropylene (Falcon) tube. Five mL of pre-heated (65°C) total RNA extraction buffer {2% (w/v) CTAB (Sigma), 2% (w/v) polyvinylpyrrolidone (PVP-40) (Sigma), 100 mM Tris HCl (pH 8.0), 25 mM EDTA, 2 M NaCl, 0.1% spermidine (Sigma) and 2% β-mercaptoethanol} was added to the powdered seeds in each tube and samples were incubated for 30 min at 65°C in a water bath. The samples were placed on a vortex every 5 minutes to help tissue disruption and RNA extraction in the buffer. After incubation, an equal volume of Chloroform: Isoamylalcohol (24:1) was added to each sample in a fume hood and samples were mixed with a vortex for 30 seconds. Thereafter the samples were centrifuged at 10,000 *g *for 20 minutes at 4°C. The aqueous supernatant (1 ml/tube) above the white phase was carefully transferred into 2.0 mL RNase-free microcentrifuge tubes and an equal volume of Chloroform: Isoamylalcohol was added, mixed with a vortex and centrifuged in a desktop centrifuge at 10,000 *g *for 10 minutes at 4°C. Without touching the white layer, the supernatant (1.0 ml) was distributed to Rnase free1.5 mL microcentrifuge tubes and 0.5 mL of 96-100% ethanol was added. The supernatant-ethanol mixture was immediately loaded onto RNA binding columns (0.75 mL/column) skipping filtration step (Qiagen RNA Mini extraction kit or any other similar kit) and spun at 10,000 *g *for 30 seconds at room temperature. Leftover samples were loaded on the same columns to process the entire sample. The kit protocol was followed in subsequent steps to wash and desalt the samples bound with the silica membrane of the column. Finally, the RNA from each column was eluted using 50 μL of RNase free water and stored at -80°C.

The quality of RNA was checked using a spectrophotometer (NanoDrop, Technologies Inc.) at two wavelength ratios of A_260/230 _and A_260/280 _nm. The integrity of total RNA was determined by running samples on 1.2% denaturing agarose gel (Qiagen, RNeasy Mini Handbook). The intensity of 28S and 18S bands was quantified with Molecular Imaging software version 5.1 (Kodak, Rochester, NY). Aliquots of RNA were stored at -80°C.

### cDNA synthesis and cDNA library construction

Poly(A)^+ ^RNA was purified from total RNA of immature jatropha seeds using the Oligotex^® ^Midi mRNA kit (Qiagen, Germany), dissolved in RNase-free water, quantified with spectrophotometer (NanoDrop, Technologies Inc.), and stored at -80°C. The cDNA library was constructed using CloneMiner™ cDNA Library Construction Kit (Invitrogen). The first strand of cDNA was synthesized using 5 μg poly(A)^+ ^RNA and converted into double strand cDNA (ds cDNA) containing *att*B sequences on each end followed by ligating *att*B1 adapter to the 5' end of cDNA. The cDNA was size fractionated by column chromatography to remove excess of primers, adapters, and small cDNAs. A non-radio labeled method was used to determine the cDNA yield. About 75-100 ng of cDNA obtained from different pooled fractions was used in site-specific recombination and *att*B-flanked cDNA was cloned into an *att*P-containing donor vector (pDONR™ 222). The BP reactions were transformed into ElectroMAX DH10B T1 phage resistant cells using an electroporator (Life technologies) and the transformed cells were plated on kanamycin (50 μg/mL) added LB agar media. Twenty positive clones were picked for verification of cDNA inserts. The mini-prepared plasmids were digested with *BsrG*1 enzyme (New England Biolabs) and electrophoresed on 1% agarose gel to determine average insert size of cDNA.

### Amplification of KAR gene using jatropha cDNA

Primers from the Arabidopsis 3-ketoacyl-acyl carrier protein reductase, (AT1G24360) (KAR) involved in Fatty acid biosynthesis were selected from the GenBank database [20] for RT-PCR on jatropha seed cDNA. The amplification program for PCR consisted of an initial denaturation step at 94°C for 2 min, followed by 35 cycles of 30 s denaturing (94°C), 45 s annealing (60°C), 1 min elongation (72°C), and a final extension at 72°C for 5 min. The amplified PCR product was visualized on agarose gel [21], and extracted from the gel using the QIAquick PCR purification kit (QIAGEN) to probe RNA blot. The PCR product was cloned into the Teasy vector (Promega) and sequenced using BigDye termination method with AB1377 sequencer (Applied Biosystems, Foster City, CA, USA). The sequenced product was confirmed by aligning with the Arabidopsis KAR nucleotide sequence at NCBI using BLAST.

### Northern blot analysis

For Northern blotting, 15 μg of RNA was isolated from immature jatropha seeds and leaves and fractionated on 1.2% agarose-formaldehyde denaturing gel (Qiagen RNeasy Mini handbook). The RNA was blotted onto Hybond-N^+ ^nylon membranes (Amersham Pharmacia) and stained for visualization of the RNA bands [17]. The KAR cDNA probe generated using RT-PCR was labelled with [32P]-dCTP (GE Healthsciences). Pre-hybridization was for 3 hours and hybridization was for 16 hours at 65°C (Techne, Staffordshire UK). Filters were washed first (20 min) in buffer A (2 × SSC + 0.1% SDS) and then Buffer B (20 min) in (1 × SSC + 0.1% SDS) and lastly (30 min) in buffer C (0.5 × SSC + 0.1% SDS) at 65°C. The bound probe was detected by exposing filters to KODAK Biomax MS Autoradiography Film using exposure cassettes at -80°C.

## Results and Discussion

**Figure 1:**
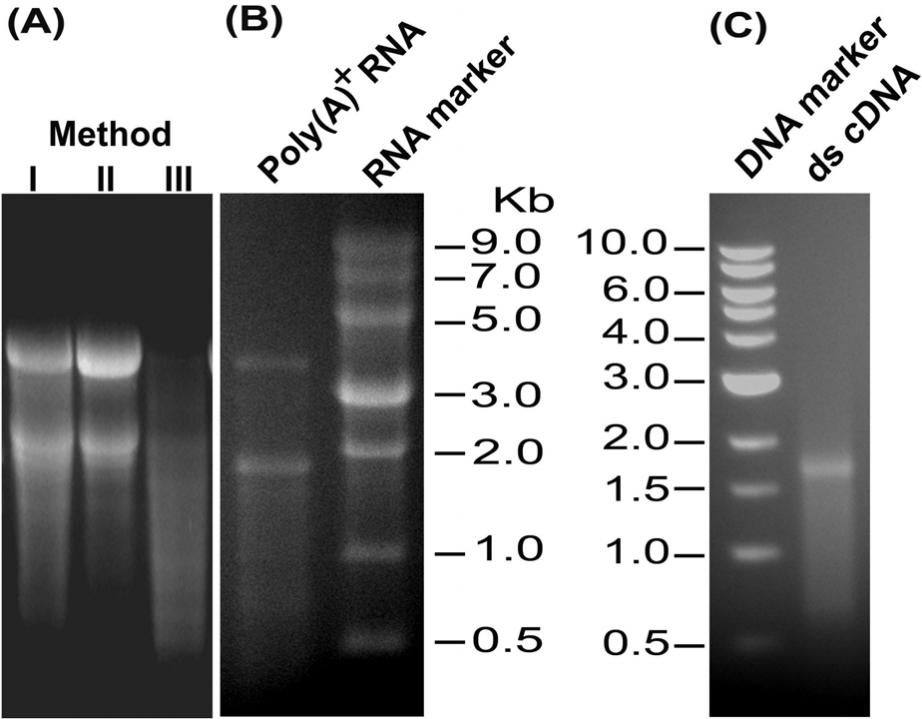
**Detection of RNA extracted with different methods, Poly(A)^+ ^RNA and the cDNA quality using agarose gel electrophoresis**. Total RNA from jatropha immature seeds: (A) RNA extracted with method I(Lane i), RNA extracted with method II (Lane ii) and RNA extracted with Qiagen RNA Mini Kit (Lane iii). (B) Poly(A)^+ ^RNA purified from the total RNA using modified method II. (C) Double strand cDNA (ds cDNA) after size fractionation with column chromatography. The RNA and 1-kb DNA marker were from New England Biolabs. The image is representative of four independent experiments.

We tried a few protocols of RNA extraction based on CTAB [5,7,8,17], acid guanidinium thiocyanate-phenol-chloroform [22] and commercial RNA extraction kits to isolate total RNA from immature jatropha seeds (data not shown), but a high yield and quality of total RNA was only achieved with the modified method (II) reported in this study (Figure 1, Table 1). The simplified method II combined the CTAB based RNA extraction with RNA binding silica columns (RNeasy^® ^Plant Mini Kit) and skipped the LiCl precipitation step to reduce the extraction time from two days to ~3 h.

**Table 1:** RNA yield and quality detected with spectrophotometer^1^

Sample	Method^2^	A_260/280_	A_260/230_	28S:18S	RNA yield (μg)/FW^3 ^(g)	Time
Jatropha	I	1.98 ± 0.03	1.91 ± 0.02	1.49 ± 0.21	124.30 ± 8.82	2 d
	II	2.14 ± 0.02	2.25 ± 0.04	1.73 ± 0.08	282.42 ± 12.91	3 h
Mustard	I	1.85 ± 0.04	1.90 ± 0.04	1.24 ± 0.08	189.95 ± 5.00	2 d
	II	2.05 ± 0.04	2.32 ± 0.01	1.68 ± 0.18	240.55 ± 11.36	3 h
Rice	I	1.94 ± 0.03	1.95 ± 0.02	1.55 ± 0.22	254.90 ± 12.74	2 d
	II	2.00 ± 0.03	2.20 ± 0.02	1.85 ± 0.03	335.35 ± 17.36	3 h

^1^Based on 4 individual samples

**Method I**, CTAB method with LiCl precipitation

^2^**Method II**, combination of CTAB based total RNA extraction method and RNeasy^® ^Plant Mini Kit (Qiagen, Germany).

FW, fresh weight,

^3^Based on Nanodrop readings.

RNA extracted with methods I (CTAB only), II (CTAB + silica column), and III (RNA extraction kit) were electrophoresed on denatured 1.2% agarose gel to determine the quality and integrity of RNA bands (Figure 1). Ribosomal RNA bands (28S and 18S) of jatropha visualized on agarose gel showed integrity of the RNA with method II (Lane ii) and the average ratio of 28S to 18S was 1.73+0.08 (Table 1) indicating least degradation which was a common problem with other methods we used. The CTAB based RNA extraction methods (including method I in this study) [5,17] meant for recalcitrant tree plant tissues were time consuming, taking almost two days for completion and the purity was also compromised as the A_260/230 _ratio was lower than 2. 00. The average 28S:18S ratio was 1.49+0.21, RNA showed smear on the gel and the bands were not sharp, indicating that the total RNA was still bound with contaminants or some degradation had occurred. Similar trends were observed with other published CTAB based methods and that involving phenol-guanidinium thiocyanate resulting in low RNA yield and quality (data not shown). The commercial kits for RNA extraction were also not successful as the silica columns were usually blocked with viscous extracts and the yield was extremely low and the quality was not good (Figure 1).

The improved RNA extraction method II is also efficient for other plant seeds rich in oil and starch as evident from the quality and quantity of RNA from mustard and rice seeds (Table 1). The ratio of A_260/230 _was higher than 2.0 for all these tissues indicating that the total RNA was of high purity without any contamination with polysaccharide compounds. Further, the A_260/280 _ratio was >2.0, indicating no contamination with proteins. The total RNA yield and both absorbance ratios for these tissues were low with method I.

The suitability of isolated RNA in downstream enzymatic procedures was also determined by constructing a cDNA library. The total RNA extracted with the modified method II produced a high quality poly(A)^+ ^RNA using Oligotex^® ^Midi mRNA Kit (Qiagen) (Figure 1, Panel B). The yield of poly(A)^+ ^RNA was about 3.8 μg per 1 mg total RNA. The poly(A)^+ ^RNA was precipitated with isopropanol and NaAc to a concentration of 1 μg/μL for first-strand cDNA synthesis. Size fractionated double-strand cDNA was visualized as a smear on the agarose gel (1.2%) with a size ranging from 0.5 kb to 5 kb (Figure 1, Panel C). Entry library carrying cDNA inserts was transformed into phage resistant *E. coli *cells (Invitrogen) and the positive clones were selected on kanamycin added LB plates. This cDNA library consisted of 1 × 10^7 ^clones, which should be enough to represent most of the genes expressed in immature jatropha seeds.

**Figure 2:**
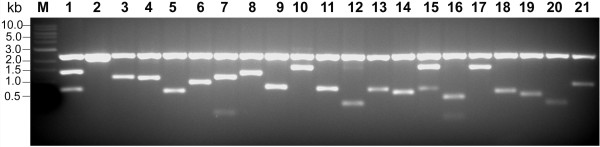
**Evaluation of jatropha seed cDNA library**. Poly(A)^+ ^RNA was purified from total RNA extracted with method II and used for cDNA library construction using CloneMiner™ cDNA Library Construction Kit (Invitrogen). Plasmid DNA of 20 positive clones was digested with *BsrG*1 enzyme (New England Biolabs) and electrophoresed on 1% agarose gel to determine average insert size of cDNA. (Lane 1) 1-kb DNA marker (New England Biolabs); (lane 2) vector pDONR™ 222 (Invitrogen); (Lanes 3-22) randomly picked cDNA clones. Band at size 2.5 Kb is the vector backbone. cDNA insert of the clone in lane 3 has similar size as that of vector backbone, which did not separate in this gel electrophoresis.

**Figure 3:**
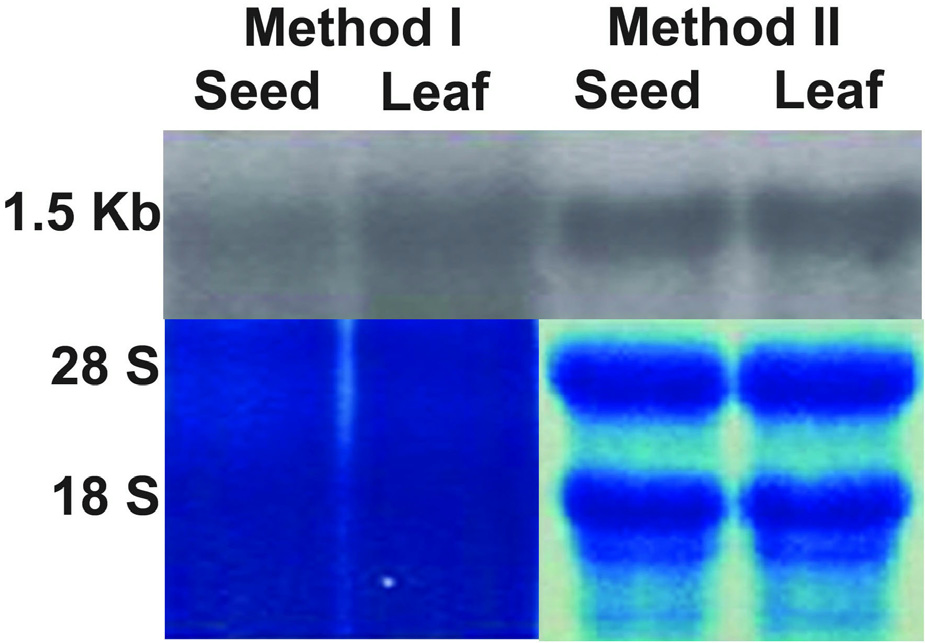
**Northern hybridization of jatropha immature seed RNA with Ketoacyl ACP reductase (KAR) probe**. RNA extracted with method I and II from jatropha leaves and seeds was fractionated on 1.2% agarose-formaldehyde denaturing gel and blotted onto Hybond-N+ nylon membrane. Stained blot was photographed and then hybridized with Ketoacyl ACP reductase (KAR) probe labelled with [32P]-dCTP. The hybridized bands were detected by exposing filters to KODAK Biomax MS Autoradiography Film at -80°C. The image is representative of four independent experiments.

To evaluate the cDNA library, plasmids were isolated from 20 randomly picked cDNA clones, digested with *Bsr*G1 enzyme (New England Biolabs) and separated on 1% agarose gel. The insert size of different cDNA clones ranged from 300 bp to 2.3 Kb with an average size of 1.3 Kb (Figure 2). DNA sequencing also indicated that all these 20 clones carried cDNA inserts (data not shown). Using primers from Arabidopsis fatty acid biosysnthesis gene, 3-ketoacyl-acyl carrier protein reductase (KAR), RT-PCR was performed on jatropha cDNA. The amplified product was sequenced and used to probe northern blot carrying jatropha leaf and seed RNA extracted with method I and II. The RNA extracted with method II showed clear bands after hybridization while smeared bands appeared with extraction method I (Figure 3). The RT-PCR product was sequenced and compared with Arabidopsis KAR gene using NCBI Blast tools that showed a 67% similarity of amino acid sequence of the coding region (data not shown).

Silica columns and silica particles have been used previously in combination with CTAB [5] based methods to improve RNA extraction from various plant tissues. CTAB-based methods were however time consuming because of the LiCl step for RNA precipitation which was eliminated in the current method to reduce the time of RNA extraction from two days to 3 hrs. In fact, when the LiCl step was used, we found some degradation of the jatropha seed RNA (data not shown). RNA extraction buffer with insoluble polyvinylpyrrolidone (PVP-40) [7] efficiently removed interfering phenolic compounds thereby preventing blockage of kit columns. As the extract was passed through the silica columns, the RNA quality was further improved in on-column cleanup process.

The information on RNA extraction protocols is limited for jatropha. An acid phenol-silica particles based method was used to extract RNA from jatropha leaf and dry seeds [19]. The method, reported as quick and effective, however needed preparation of silica particles that took about 24 h and the yield was also low. The method also required the use of toxic acid phenol for extracting RNA that has safety issues in handling and disposal. If improperly removed, RNA-phenol residual complex could interfere with reverse transcription reactions, smear on denaturing agarose gel and disturb RNA migration [23]. The method did not show any evidence for cDNA library construction or other downstream applications with jatropha RNA. Since phenol was not used in current procedure, the RNA was highly suitable for several downstream applications. The average RNA yield from the current method (282.42 ± 12.91 μg/g FW) and the band quality and ratio of 28S and 18S intensity is also good. Moreover, this method is straightforward in application as the supernatant is directly loaded on to the commercial silica columns, which is more convenient to work with.

## Conclusion

The modified protocol is simple and highly effective for extracting good quality RNA from oil rich immature jatropha seeds as well as mustard and rice seeds. It should be equally useful for other tree plants for molecular characterization where the quality and quantity of RNA is highly dependent on the presence of lipids/fatty acids, polysaccharides and polyphenols.

## Competing interests

The authors declare that they have no competing interests.

## Authors' contributions

JSS conducted the experiments. JK drafted the manuscript. JSS and KG constructed cDNA library. ZY supervised and drafted the manuscript. All authors have read and approved the final manuscript.
